# 1-Kestose supplementation mitigates the progressive deterioration of glucose metabolism in type 2 diabetes OLETF rats

**DOI:** 10.1038/s41598-020-72773-2

**Published:** 2020-09-24

**Authors:** Ayako Watanabe, Yoshihiro Kadota, Rina Kamio, Takumi Tochio, Akihito Endo, Yoshiharu Shimomura, Yasuyuki Kitaura

**Affiliations:** 1 Laboratory of Nutritional Biochemistry, Department of Applied Biosciences, Graduate School of Bioagricultural Sciences, Nagoya University, Nagoya, Aichi Japan; 2 B Food Science Co., Ltd, Chita, Aichi Japan; 3 Department of Food, Aroma and Cosmetic Chemistry, Faculty of Bioindustry, Tokyo University of Agriculture, Abashiri, Hokkaido Japan; 4 Department of Food and Nutritional Sciences, College of Bioscience and Biotechnology, Chubu University, Kasugai, Aichi Japan

**Keywords:** Endocrinology, Gastroenterology

## Abstract

The fructooligosaccharide 1-kestose cannot be hydrolyzed by gastrointestinal enzymes, and is instead fermented by the gut microbiota. Previous studies suggest that 1-kestose promotes increases in butyrate concentrations in vitro and in the ceca of rats. Low levels of butyrate-producing microbiota are frequently observed in the gut of patients and experimental animals with type 2 diabetes (T2D). However, little is known about the role of 1-kestose in increasing the butyrate-producing microbiota and improving the metabolic conditions in type 2 diabetic animals. Here, we demonstrate that supplementation with 1-kestose suppressed the development of diabetes in Otsuka Long-Evans Tokushima Fatty (OLETF) rats, possibly through improved glucose tolerance. We showed that the cecal contents of rats fed 1-kestose were high in butyrate and harbored a higher proportion of the butyrate-producing genus *Anaerostipes* compared to rats fed a control diet. These findings illustrate how 1-kestose modifications to the gut microbiota impact glucose metabolism of T2D, and provide a potential preventative strategy to control glucose metabolism associated with dysregulated insulin secretion.

## Introduction

Diabetes is considered to be a global epidemic^1^. Type 2 diabetes (T2D), which represents more than 90% of diabetic patients, is a progressive metabolic disorder characterized by insulin resistance and its onset is attributed to genetic and lifestyle factors (i.e., physical activities and diet). Further, the gut microbiota has recently been acknowledged to be a lifestyle factor, since it is highly dependent on the host diet^2^. The relative abundance of phylum Firmicutes and class Clostridia was significantly reduced in T2D adults compared to non-diabetic adults^3^. Analyses of the fecal samples of Chinese T2D patients revealed the enrichment of pathogenic species such as *Bacteroides caccae*, whereas several types of butyrate-producing species including *Faecalibacterium* and *Roseburia* were abundant in non-diabetic controls^4^. In 70-year-old European women, the metagenomic clusters (sets of genes with high correlation) of the fecal microbiota showed significant depletion of butyrate-producing species in T2D^5^. Another study reported that when fecal samples of T2D patients, whose clinical parameters were improved by treatment with high-fiber diets, were transplanted into mice, the animals mirrored the improved metabolic status of these patients^6^. Further, metagenomic analyses of fecal samples of the mice demonstrated the abundance of microbial genes associated with butyrate-producing pathways. These reports emphasize the link between T2D and the gut microbiota, especially for butyrate-producing bacteria.

Prebiotics, first introduced by Gibson and Roberfroid in 1995, are indigestible food components that stimulate the growth and/or activity of one or a limited number of resident bacterial species in the colon and thus have beneficial effects on host health^7^. Fructooligosaccharides (FOSs) are indigestible and commercially available as a prebiotic. The chemical structure of FOSs consists of a chain of fructose units with a terminal glucose unit linked by a β-2–1 osidic linkage, meaning that they cannot be hydrolyzed by gastrointestinal digestive enzymes^7^. The length of short-chain FOSs ranges from three to five molecules, consisting of 1-kestose, nystose and 1-fructosylnystose. FOSs are well known to increase the abundance of the genus *Bifidobacterium*^7^. High-fat diet-induced diabetic mice showed a restored abundance of *Bifidobacterium* spp. following supplementation with FOSs, in contrast to diabetic mice fed a high-fat diet alone^8^. Furthermore, the restoration of *Bifidobacterium* spp. showed a positive correlation with improved glucose tolerance^8^. However, the component of FOSs responsible for beneficial effects on T2D metabolic status remains to be identified.

In our previous study, supplementation with 1-kestose enhanced the growth of beneficial gut microbiota, i.e., bifidobacteria, in the cecal samples of rats^9^. Further, the levels of acetate, butyrate and lactate in the cecal contents were markedly elevated in a 1-kestose dose-dependent manner^9^. Furthermore, in vitro, the butyrate producer *Anaerostipes caccae* preferentially consumed 1-kestose in medium contained other FOS components, i.e., nystose and 1-fructosylnystose^10^.

In order to determine whether 1-kestose might have beneficial effects on T2D metabolic status, Otsuka Long-Evans Tokushima Fatty (OLETF) rats, which are a T2D model, were fed the 1-kestose supplemented diet for 16 weeks. OLETF rats induce a diabetic status by overfeeding, resulting in moderate onset of hyperglycemia^11^. Our study revealed that supplementation with 1-kestose mitigated development of glucose intolerance and modified plasma insulin levels in T2D model rats, in association with increased cecal butyrate concentrations via alterations in microbiota communities.

## Results

### Body weight, food intake, and water intake

The final body weights tended to be higher in OLETF rats than in LETO rats fed either CON or KES (P = 0.39 or P = 0.19, respectively), and little difference was observed between CON and KES groups of both rats (Table 1). Average food intake showed the same tendency as final body weight; OLETF rats showed a higher intake than LETO rats, and the highest intake was in OKETF/KES among the four groups (Table 1). OLETF/CON displayed an about three-fold increase of average water intake during 22–23 weeks of age compared to LETO/CON, however the water intake was not significantly different between OLETF/KES and OLETF/CON (P = 0.83) (Table 1).

**Table 1:** Rat body weight, and food and water intakes.

Item	LETO		OLETF	
	CON	KES	CON	KES
Body weight (g)^a^	619 ± 23	626 ± 27	666 ± 18	686 ± 13
Food intake (g/day)^b^	27.1 ± 0.7	30.5 ± 1.5	32.8 ± 0.8^#^	37.5 ± 0.5^$^^,^*
Water intake (g/day)^c^	27.0 ± 1.7	23.2 ± 1.7	72.4 ± 14.5^#^	60.3 ± 9.6

Values represent the mean ± SEM, n = 5–8.

*LETO* Long-Evans Tokushima Otsuka; *OLETF* Otsuka Long-Evans Tokushima Fatty; *CON* control diet; *KES* 1-kestose diet.

^#^P < 0.05 compared with LETO/CON.

^$^P < 0.05 compared with LETO/KES.

*P < 0.05 compared with OLETF/CON. The data were compared using Tukey–Kramer’s test.

^a^Body weight is a value at the termination of the experiment.

^b^Food intake is the average of the 16 weeks of the experimental period.

^c^Water intake is the average of one week of the recorded period from age 22 to 23-week-old (from 15 to 16th week of the experiment).

### Fasting plasma glucose and insulin concentrations

**Figure 1:**
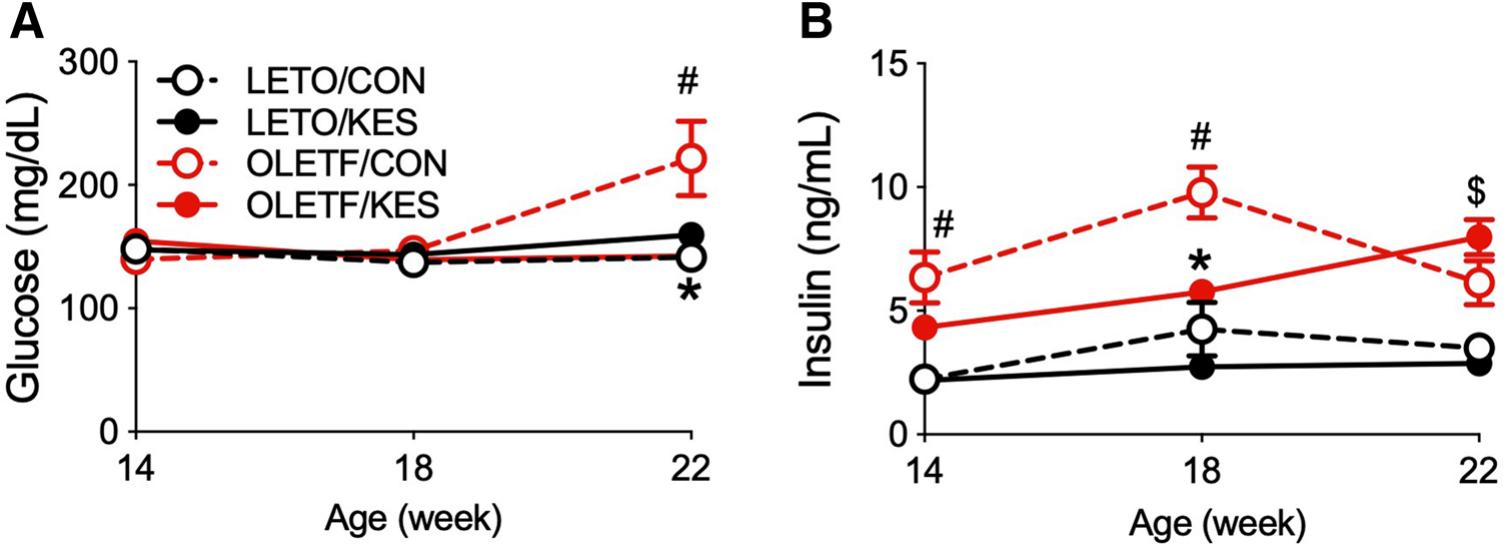
Concentrations of fasting plasma glucose (**A**) and insulin (**B**) at 14, 18 and 22 weeks of age in LETO and OLETF rats. Data are shown as the mean ± SEM, n = 5–8. ^#^P < 0.05 compared with LETO/CON, ^$^P < 0.05 compared with LETO/KES, *P < 0.05 compared with OLETF/CON. The data were compared using Tukey–Kramer’s test at each time point. *LETO* Long-Evans Tokushima Otsuka; *OLETF* Otsuka Long-Evans Tokushima Fatty; *CON* control diet; *KES* 1-kestose diet.

To assess the effects of 1-kestose supplementation on glucose metabolism in the T2D model, we measured fasting plasma glucose and insulin concentrations over the course of the experimental period in OLETF rats. The fasting plasma glucose concentrations in LETO and OLETF rats were not different up to 18 weeks of age (Fig. 1A). At 22 weeks of age, the fasting plasma glucose concentration was elevated only in OLETF/CON, and that in OLETF/KES was comparable to that in LETO rats; thus, values were significantly different between OLETF/CON and OLETF/KES (Fig. 1A).

At 14 weeks of age, fasting plasma insulin concentrations tended to be higher in OLETF rats than that in LETO rats; however, only that in OLETF/CON was significantly higher than that in LETO rats (Fig. 1B). At 18 weeks of age, the insulin concentration in OLETF/CON was further elevated, but that in OLETF/KES remained almost constant; thus, the insulin concentration was significantly higher only in OLETF/CON than the other three groups (Fig. 1B). At 22 weeks of age, the insulin concentration in OLETF/CON declined and was not significantly different from that in LETO rats (Fig. 1B). On the other hand, the insulin concentration in OLETF/KES at the same age was elevated and was significantly higher than that in LETO/KES (Fig. 1B).

### C-peptide concentration

**Figure 2:**
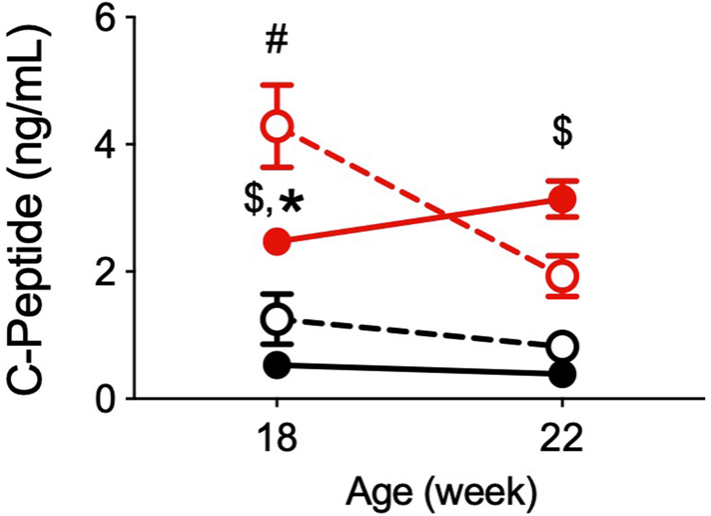
Plasma C-peptide concentrations at 18 and 22 weeks of age in LETO and OLETF rats. Data are shown as the mean ± SEM, n = 5–8. ^#^P < 0.05 compared with LETO/CON, ^$^P < 0.05 compared with LETO/KES, *P < 0.05 compared with OLETF/CON. The data were compared using Tukey–Kramer’s test at each time point. *LETO* Long-Evans Tokushima Otsuka; *OLETF* Otsuka Long-Evans Tokushima Fatty; *CON* control diet; *KES* 1-kestose diet.

Since the decrease in insulin concentration in OLETF/CON at 22 weeks of age suggests a decline in insulin secretion from pancreatic β-cells^12^, we measured the C-peptide concentration at 18 and 22 weeks of age. The C-peptide concentrations of the four groups at both 18 and 22 weeks of age and the observed changes from 18 and 22 weeks of age showed almost the same pattern as for insulin concentrations (Fig. 2), suggesting that insulin secretion declined only in OLETF/CON.

### Glycemic response

**Figure 3:**
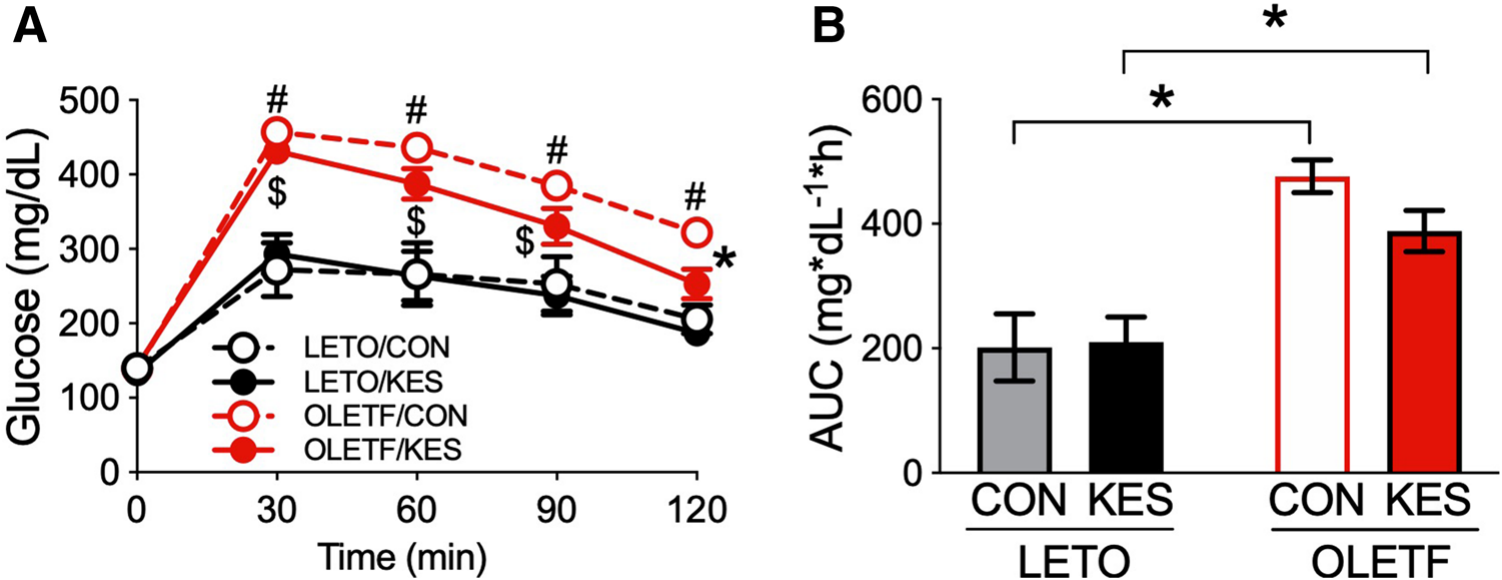
OGTT in LETO and OLETF rats. Results of the glucose tolerance test (**A**) were assessed in LETO and OLETF rats fed CON and KES at 20 and 21 weeks of age. The area under the curve (**B**) was measured between 0 and 120 min after glucose load. Data are shown as the mean ± SEM, n = 5–8. ^#^P < 0.05 compared with LETO/CON, ^$^P < 0.05 compared with LETO/KES, *P < 0.05 compared with OLETF/CON (**A**). *P < 0.05 compared with the control diet groups of both rats (**B**). The data were compared using Tukey–Kramer’s test. *LETO* Long-Evans Tokushima Otsuka; *OLETF* Otsuka Long-Evans Tokushima Fatty; *CON* control diet; *KES* 1-kestose diet; *AUC* area under the curve.

We evaluated the effect of 1-kestose supplementation on glucose tolerance in OLETF rats. In the OGTT, plasma glucose concentrations were higher in OLETF rats than in LETO rats (Fig. 3A). Although 1-kestose supplementation had no effect on the patterns of plasma glucose concentrations in LETO rats, the plasma glucose concentrations in OLETF rats tended to be lower in the 1-kestose group, and the concentration at 120 min in the OGTT was significantly lower in OLETF/KES than in OLETF/CON (Fig. 3A).

Values of the area under the curve (AUC) for plasma glucose were significantly higher in OLETF rats than in LETO rats (Fig. 3B). Although there were no significant differences in the AUC between control and 1-kestose diet groups of either rat type, plasma glucose tended to be lower in OLETF/KES than in OLETF/CON (P = 0.27) (Fig. 3B).

### Tissue weights and concentrations of blood components

To examine whether 1-kestose supplementation imparts physiological effects on the study groups, we compared tissue weights and concentrations of blood components. We found that the liver and perirenal adipose tissue weight were higher in OLETF rats than in LETO rats after 16 weeks of the experimental diet (Table 2). Yet, there were no significant differences between the two dietary groups in either OLETF or LETO rats (Table 2).

**Table 2:** Rat tissue weights.

Tissue	LETO		OLETF	
	CON	KES	CON	KES
	(g)			
Liver	19.9 ± 0.8	21.1 ± 1.0	30.3 ± 0.7^#^	31.3 ± 1.2^$^
Heart	1.38 ± 0.02	1.36 ± 0.06	1.47 ± 0.04	1.50 ± 0.03
**Adipose tissue**				
Perirenal	23.8 ± 3.6	22.6 ± 2.2	47.8 ± 2.6^#^	42.6 ± 1.9^$^
Epididymal	18.4 ± 1.7	19.0 ± 1.9	22.2 ± 0.9	23.6 ± 0.6

Values represent the mean ± SEM, n = 5–8.

*LETO* Long-Evans Tokushima Otsuka; *OLETF* Otsuka Long-Evans Tokushima Fatty; *CON* control diet; *KES* 1-kestose diet.

^#^P < 0.05 compared with LETO/CON.

^$^P < 0.05 compared with LETO/KES. The data were compared using Tukey–Kramer’s test.

Blood samples were collected under a fed condition at the end of the experiment. The concentrations of all blood components measured, except for insulin, were significantly higher in OLETF rats than in LETO rats; however, they were not different between the two dietary groups in either OLETF or LETO rats (Table 3). Consistent with the values of OLETF/CON and OLETF/KES in Figs. 1B and 2, plasma insulin levels tended to be lower in OLETF/CON than in OLETF/KES, although the difference was not statistically significant (P = 0.88) (Table 3).

**Table 3:** Concentrations of blood components.

Component	LETO		OLETF	
	CON	KES	CON	KES
Glucose (mg/dL)	188 ± 12.7	199 ± 10.5	604 ± 53.2^#^	577 ± 31.5^$^
Insulin (ng/mL)	10.2 ± 3.1	8.1 ± 1.4	5.4 ± 1.8	7.2 ± 1.5
Triglyceride (mg/dL)	138 ± 10.3	122 ± 15.2	319 ± 30.9^#^	361 ± 19.2^$^
Free fatty acids (µEQ/L)	500 ± 30.2	527 ± 41.0	976 ± 80.8^#^	998 ± 66.6^$^
Total cholesterol (mg/dL)	144 ± 3.8	141 ± 4.0	204 ± 5.4^#^	198 ± 7.6^$^
HDL (mg/dL)	28.6 ± 0.7	28.7 ± 0.5	36.9 ± 0.9^#^	36.4 ± 1.0^$^
LDL (mg/dL)	15.0 ± 0.8	14.0 ± 0.4	25.1 ± 1.0^#^	23.0 ± 1.0^$^

Values represent the mean ± SEM, n = 5–8.

*LETO* Long-Evans Tokushima Otsuka; *OLETF* Otsuka Long-Evans Tokushima Fatty; *CON* control diet; *KES* 1-kestose diet; *HDL* high-density lipoprotein; *LDL* low-density lipoprotein.

^#^P < 0.05 compared with LETO/CON.

^$^P < 0.05 compared with LETO/KES. The data were compared using Tukey–Kramer’s test.

### Weights of ceca and cecal contents, and cecal pH

**Figure 4:**
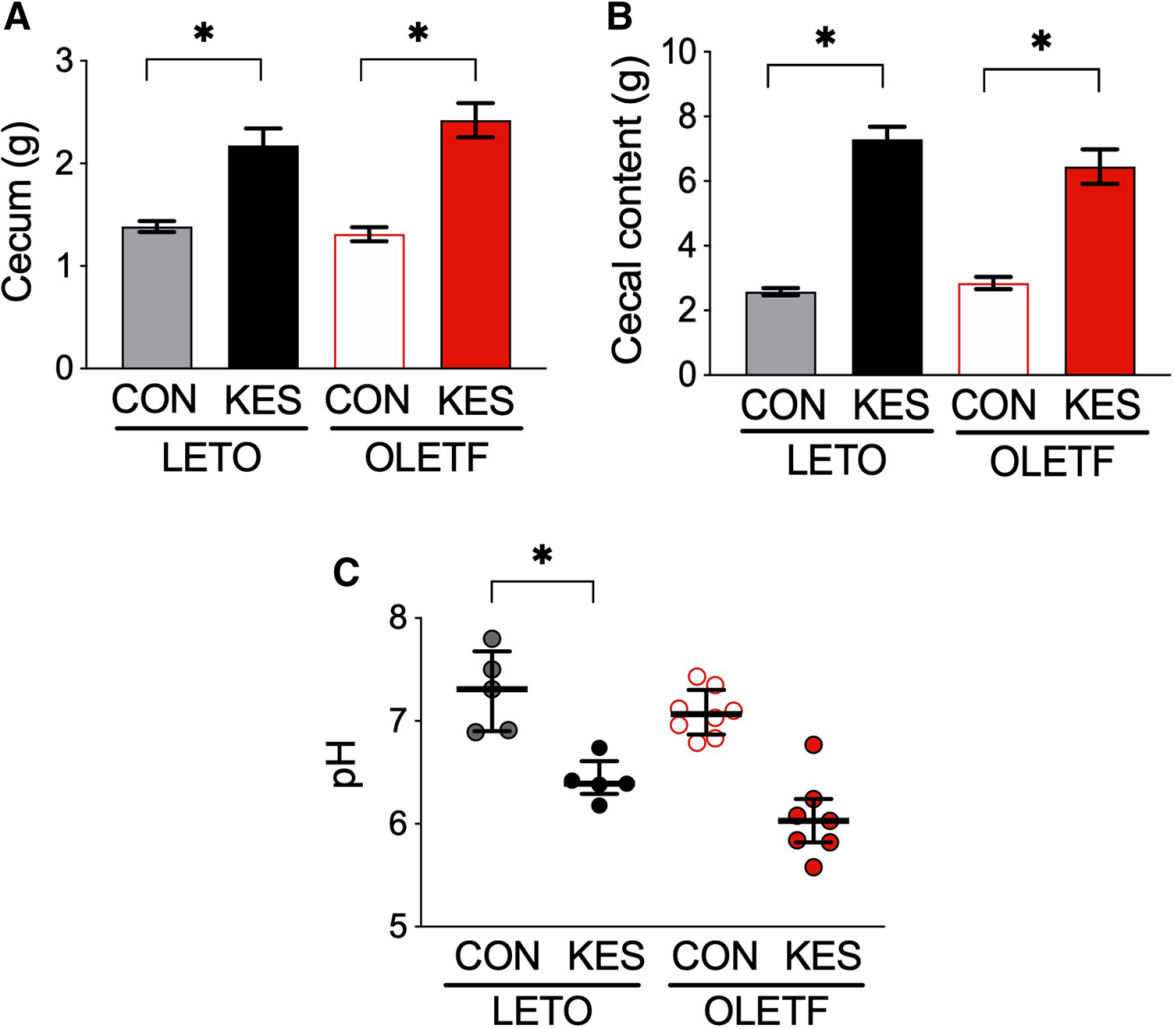
Weights of ceca (**A**) and cecal contents (**B**), and cecal pH (**C**) at the end of the experiment in LETO and OLETF rats fed CON and KES. The data are shown as the mean ± SEM, n = 5–8 (**A**,**B**). The data in (**C**) are expressed as a scatter dot plot with a horizontal line indicating the median value plus the interquartile range. *P < 0.05 compared with the control diet of both rats. The data were compared using Tukey–Kramer’s test. *LETO* Long-Evans Tokushima Otsuka; *OLETF* Otsuka Long-Evans Tokushima Fatty; *CON* control diet; *KES* 1-kestose diet.

At the end of the experiment, we assessed the effect of 1-kestose on the intestinal environment. 1-Kestose supplementation induced an about two-fold increase in the cecal weights of LETO and OLETF rats (Fig. 4A). Increases in the weights of cecal contents were also observed with 1-kestose supplementation in both rats (Fig. 4B). The pH of cecal contents tended to be lower in the KES groups than in the CON groups of both rats, indicating the slight acidification of cecal contents in the KES groups of both rats (OLETF/KES versus OLETF/CON, P = 0.19) (Fig. 4C). These results suggest that 1-kestose supplementation alters the gut environment, presumably via microbial metabolites such as SCFAs and changes in microbiota communities.

### SCFA composition in cecal contents

**Figure 5:**
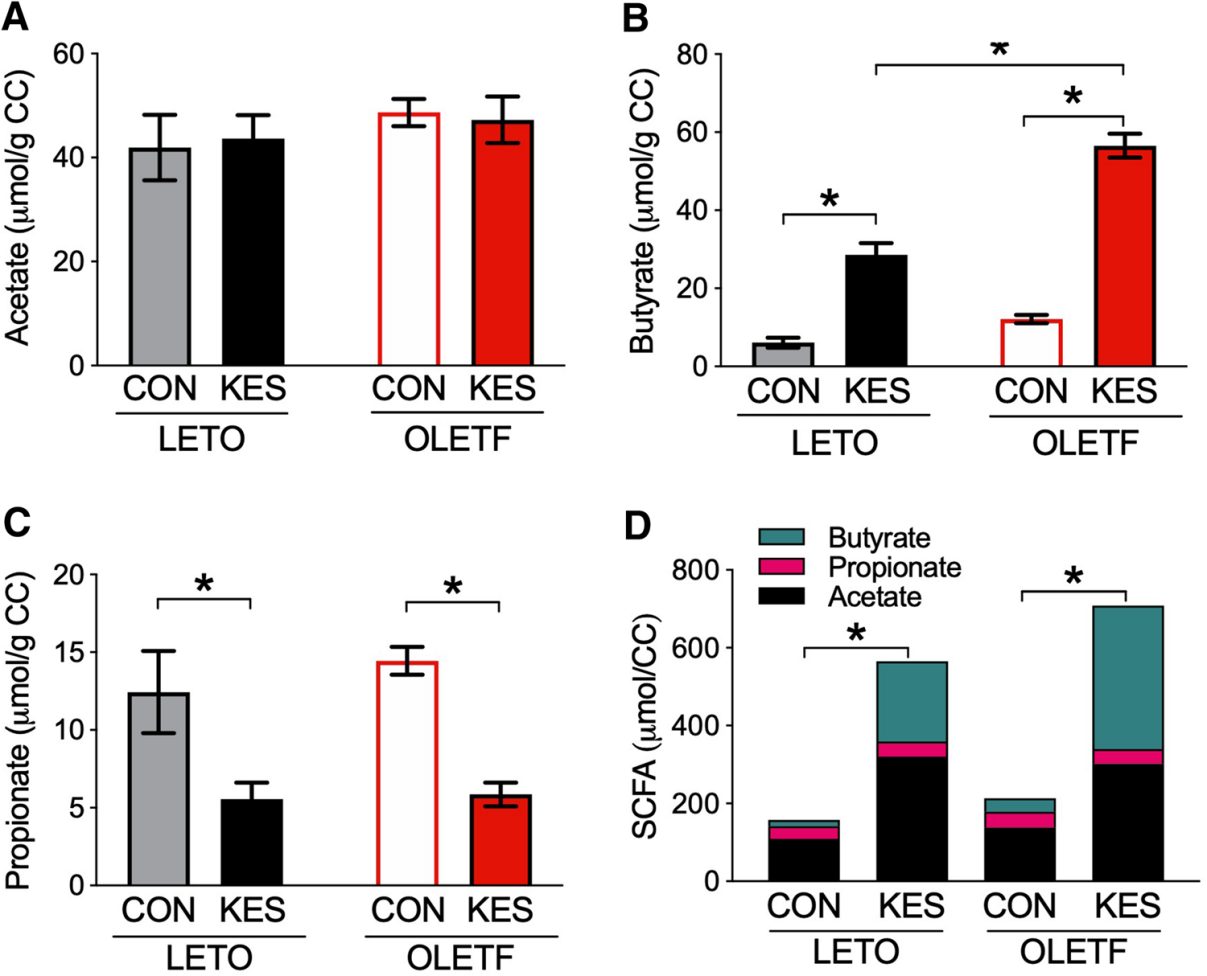
Enhancement of total SCFAs in the cecal contents of LETO/KES and OLETF/KES. Concentrations of acetate (**A**), butyrate (**B**) and propionate (**C**) were measured in the cecal contents of LETO and OLETF rats fed CON and KES. Total amount of SCFAs in the ceca of each group (**D**). Data are shown as the mean ± SEM, n = 5–8. *P < 0.05, by Tukey–Kramer’s test. *LETO* Long-Evans Tokushima Otsuka; *OLETF* Otsuka Long-Evans Tokushima Fatty; *CON* control diet; *KES* 1-kestose diet; *SCFAs* short-chain fatty acids; *CC* cecal content.

Supplementation with 1-kestose may exert its effects on glucose metabolism through increases in SCFA production in the cecum. Concentrations of acetate in the cecal contents were comparable among all groups (Fig. 5A), but the total amount of acetate was increased in the cecum of LETO/KES and OLETF/KES (P < 0.05) (Fig. 5D). Concentrations of butyrate in the cecal contents and total amounts in the cecum were greatly enhanced in LETO/KES and OLETF/KES compared to LETO/CON and OLETF/CON, respectively (total butyrate amount, P < 0.05) (Fig. 5B,D). Although cecal concentrations of propionate were reduced by 1-kestose supplementation in both rats (Fig. 5C), the total amounts were unchanged (Fig. 5D). Cecal contents of valerate, isobutyrate, and isovalerate tended to be lower in the KES groups than in the CON groups of both rats; however, those trends were inverted due to increased cecal content weights in the KES groups (Table 4).

**Table 4:** Concentrations and total amounts of minor short-chain fatty acids in cecal contents.

Short-chain fatty acid	LETO		OLETF	
	CON	KES	CON	KES
	Concentration (µmol/g cecal content)			
Valerate	1.53 ± 0.20	0.98 ± 0.11^†^	1.95 ± 0.11	1.04 ± 0.04*
Isobutyrate	1.23 ± 0.19	0.78 ± 0.09	1.60 ± 0.08	0.91 ± 0.09*
Isovalerate	1.10 ± 0.14	0.76 ± 0.07	1.39 ± 0.09	1.00 ± 0.14
	Total amount (µmol/ cecal content)			
Valerate	3.94 ± 0.58	6.95 ± 0.66^†^	5.53 ± 0.45	6.80 ± 0.77
Isobutyrate	3.16 ± 0.52	5.48 ± 0.45	4.58 ± 0.40	5.96 ± 0.96
Isovalerate	2.82 ± 0.37	5.41 ± 0.44	3.91 ± 0.29	6.54 ± 1.09*

Values represent the mean ± SEM, n = 5–8.

*LETO* Long-Evans Tokushima Otsuka; *OLETF* Otsuka Long-Evans Tokushima Fatty; *CON* control diet; *KES* 1-kestose diet.

^†^P < 0.05 compared with LETO/CON.

*P < 0.05 compared with OLETOF/CON. Data were compared using Tukey–Kramer’s test.

### Gut microbial composition of the ceca

**Figure 6:**
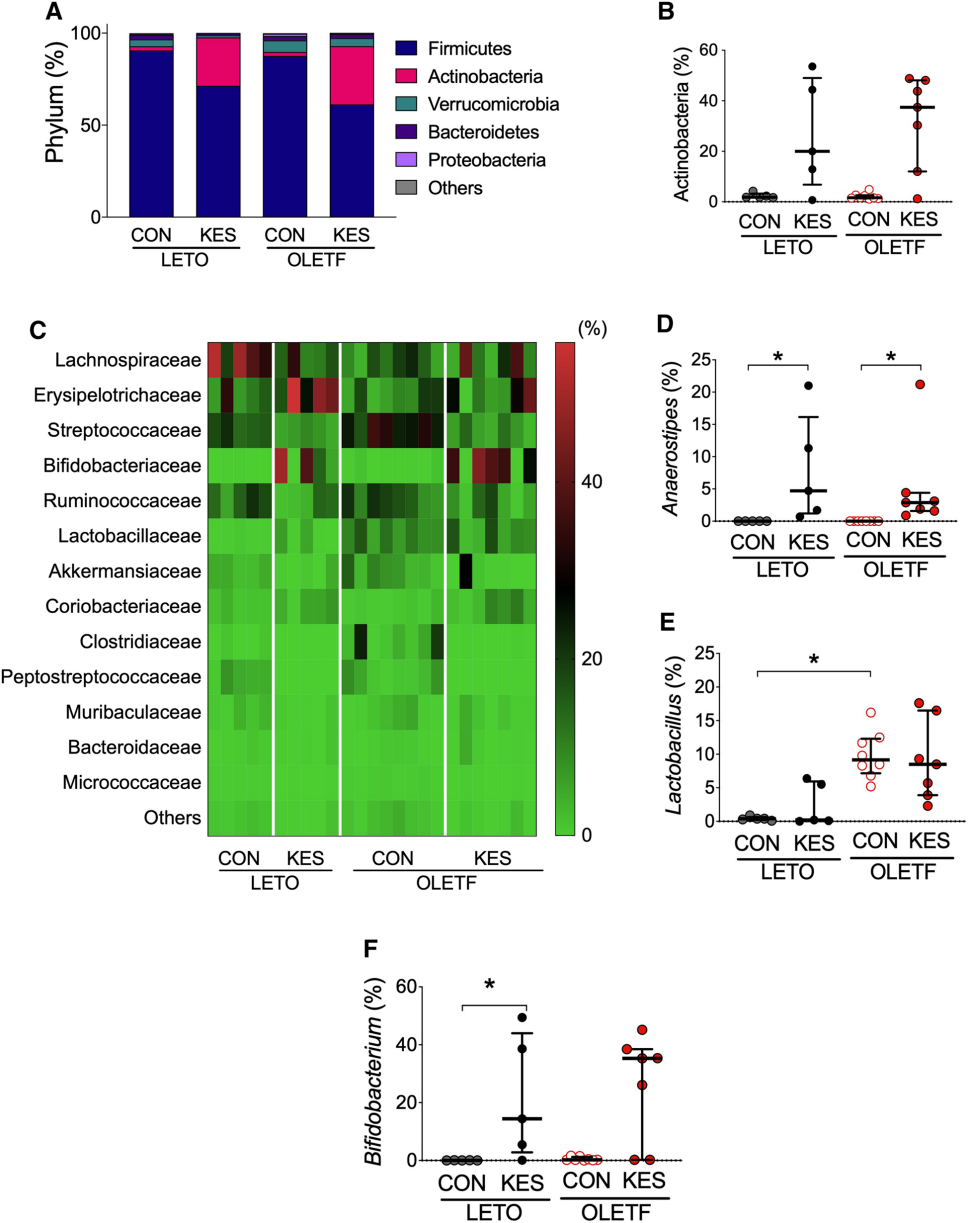
Alterations in gut microbial composition with 1-kestose supplementation. Compositional analysis of the microbiota was conducted by 16S rRNA sequencing of LETO and OLETF rats fed CON and KES (n = 5–8). The relative abundance of major phyla (**A**), Actinobacteria (**B**) and the indicated family (**C**). The bar charts show the abundance of each phylum (**A**). The relative abundance of the indicated family was displayed as a heatmap (**C**). The relative abundance of the genera *Anaerostipes* (**D**), *Lactobacillus* (**E**) and *Bifidobacterium* (**F**). The data in (**B**,**D**–**F**) are expressed as a scatter dot plot with a horizontal line indicating the median value plus the interquartile range. *P < 0.05, by Kruskal–Wallis test followed by Dunn’s multiple comparison test. *LETO* Long-Evans Tokushima Otsuka; *OLETF* Otsuka Long-Evans Tokushima Fatty; *CON* control diet; *KES* 1-kestose diet.

Finally, to determine the effect of gut microbiota composition on alterations of SCFA components, 16S rRNA gene sequencing was performed to study the gut microbiota of cecal samples collected from the experimental groups. The most abundant phylum was Firmicutes in all groups (Fig. 6A). Cecal samples of LETO/KES and OLETF/KES showed markedly increased relative abundance (mean) of Actinobacteria from 2.3 to 26.3% of OTUs and from 2.2 to 31.7% of OTUs, respectively, compared to their counterparts (Fig. 6A,B). At a family level, a relatively lower abundance of Lachnospiraceae, belonging to the phylum Firmicutes*,* was observed in OLETF/CON compared to LETO/CON (P = 0.06) (Fig. 6C). The abundance of Lactobacillaceae was higher in OLETF/CON compared to LETO/CON (P < 0.05) (Fig. 6C). Focusing on specific genera within Lachnospiraceae, *Anaerostipes* with some represented by butyrate-producing bacteria, was present at significantly higher levels in LETO/KES and OLETF/KES compared to LETO/CON and OLETF/CON (Fig. 6D). In accordance with the higher abundance of the family Lactobacillaceae in OLETF/CON, *Lactobacillus* was observed at a significantly higher level compared to LETO/CON, whereas OLETF/KES showed no significant difference in the genus from LETO/KES (Fig. 6E). Consistent with the higher level of the family Bifidobacteriaceae in LETO/KES and OLETF/KES compared to their counterparts (P < 0.05 and P = 0.30, respectively), the level of the genus *Bifidobacterium* was higher in the cecal contents of LETO/KES, and showed a higher trend in OLETF/KES (P = 0.30) compared to its counterpart (Fig. 6C,F). At a species level, *A. caccae* was found at significantly higher levels in LETO/KES and OLETF/KES compared to their counterparts (Table 5). 1-Kestose supplementation in OLETF rats tended to lower the abundance of *Akkermansia muciniphila* compared to OLETF/CON (P = 0.06) (Table 5). Taken together, 1-kestose induced the accumulation of butyrate by increasing the level of the butyrate-producing microbe *A. caccae*.

**Table 5:** Relative abundance of each species.

Species	LETO		OLETF	
	CON	KES	CON	KES
	(% of read)			
*Anaerostipes caccae*	0.00 (0.00–0.01)	4.61^†^ (1.65–16.17)	0.00 (0.00–0.00)	2.85* (1.76–4.45)
*Akkermansia muciniphila*	4.60 (2.34–4.67)	0.33 (0.00–2.73)	5.31 (3.01–9.59)	0.32 (0.01–1.62)

Values represent the median value plus the interquartile range, n = 5–8.

*LETO* Long-Evans Tokushima Otsuka; *OLETF* Otsuka Long-Evans Tokushima Fatty; *CON* control diet; *KES* 1-kestose diet.

^†^P < 0.05 compared with LETO/CON.

*P < 0.05 compared with OLETOF/CON. Data were compared using Kruskal–Wallis test followed by Dunn’s multiple comparison test.

## Discussion

In the present study, we investigated the effects of 1-kestose supplementation on glucose metabolism and gut microbiota in a T2D animal model. We used OLETF rats as the T2D model, and they displayed relatively slow development of insulin resistance and hyperglycemia compared to other genetically obese and diabetic model animals, such as Zucker fatty rats and ob/ob mice. As such, the effects of prebiotics on the gut microbiota may be less acute^13^. OLETF rats fed a control diet (OLETF/CON) in this study showed hyperinsulinemia at 14 and 18 weeks of age without hyperglycemia (Fig. 1B). At 22 weeks of age, they then became hyperglycemia with decreased insulin level due to the decline in insulin secretion from pancreatic β-cells, resulting in increased water intake, a typical symptom of diabetes (Figs. 1A and 2; Table 1). In contrast, 1-kestose supplementation resulted in later onset of these glycemic symptoms in OLETF rats; the insulin level in OLETF/KES was significantly higher than that in LETO/KES only at 22 weeks of age (Fig. 1B), but the blood glucose level in OLETF/KES remained constant at a level similar to that observed in the LETO rats (Fig. 1A). Following 16-week 1-kestose supplementation in both LETO and OLETF rats, the ceca were enlarged (Fig. 4A), and cecal butyrate concentrations were markedly increased (Fig. 5B). Also, cecal valerate and isovalerate levels were increased in LETO/KES and OLETF/KES, respectively (Table 4). These results suggest that the alteration of SCFAs, specifically butyrate, in the ceca may be responsible for a suppressive effect on glucose intolerance in OLETF/KES.

The results of the present study indicate the potential of 1-kestose to delay T2D onset and reduce glucose intolerance severity, which is supported by the previous reports using FOSs^8,13^. Glucose tolerance was improved by either 13 weeks of supplementation with 10% FOSs to a high-fat diet^8^ or six weeks of FOS-added water intake^13^ in diet-induced diabetic mice with showing the reduced body weight. Our study did not affect body weight (Table 1), which may be attributable to differences in experimental conditions, particularly, the duration of the experiment investigating the chronic effects of 1-kestose on T2D onset. One possible mechanism behind the suppressive effect of 1-kestose on glucose intolerance may be the involvement of incretin hormone glucagon-like peptide-1 (GLP-1). Plasma GLP-1 concentrations were increased by indigestible carbohydrate intake in rodents and T2D subjects, co-occurring with improved glucose tolerance or postprandial hyperglycemia^13–15^, and the increased GLP-1 secretion was due to SCFAs produced by the gut microbiota through metabolism of the indigestible carbohydrates^16^. These findings imply that GLP-1 secretion participated in the reduction of glucose intolerance severity observed in OLETF/KES through an increased SCFA production by 1-kestose metabolism of gut microbiota (Fig. 5). This possibility may be clarified by a further detailed study examining the acute and chronic effects of 1-kestose supplementation on plasma incretin levels.

In this study, 1-kestose supplementation increased the cecal weight and lowered pH levels and was accompanied by a remarkable increase in total SCFA concentrations (Figs. 4 and 5), as described by others^7,17^. In our previous study, supplementation with 1-kestose also raised the levels of acetate and butyrate in the cecal contents of Sprague–Dawley (SD) rats, while there was no significant difference in the level of propionate between the control and 1-kestose supplemented groups^9^. The conflicting results of 1-kestose supplementation on SCFA production between the previous and present works might be attributable to differences in animal species (SD versus LETO and OLETF) and duration of feeding period (4 versus 16 weeks), which might change the gut environment of the animals, especially pH; the acidic pH 5.5 preferentially promotes butyrate production and limits propionate formation in vitro^18,19^. Thus, our findings suggest that the high production of butyrate, in response to 1-kestose supplementation, may lead to increasing gut acidity, and provide a more favorable gut environment for butyrate producers.

We observed a higher proportion of butyrate-producing *A. caccae* in rats fed KES than in those fed CON (Fig. 6D; Table 5). Consistent with our previous in vitro study^10^, the present study indicates that feeding 1-kestose stimulates the growth of *A. caccae *in vivo, resulting in a marked accumulation of butyrate. The microorganism is also known to generate ATP from lactate as a carbon source, which leads to the accumulation of butyrate^20^. In addition, we demonstrated that 1-kestose increased the proportion of bifidobacteria in the rat ceca (Fig. 6F). Bifidobacteria produce lactate and acetate via carbohydrate metabolism, although the ratio of the two acids produced varies in accordance with the available carbohydrates^10^. This might suggest that bifidobacteria metabolized 1-kestose and produced lactate and acetate. The lactate produced was presumably a source of butyrate production by *A. caccae* in the ceca of LETO/KES and OLETF/KES. Similar findings were reported by feeding galactooligosaccharides and *A. caccae* in rats, and by administration of germinated barley foodstuff in humans^20–22^. Previous studies revealed that this species grows well on degrees of polymerization (DP) 3 FOS, 1-kestose, but shows poor growth on DP4 FOS, nystose. This difference in growth stimulation activities is due to substrate specificities of FOS degradation enzymes in the organism and gene induction activity attributable to the two FOSs^10,23^. These findings indicate that beneficial physiological outcomes such as suppressing the progress of T2D observed in KES-fed rats, are specific to certain oligosaccharides including 1-kestose. A recent study revealed that *A. caccae* was protective against an allergic response to food^24^, which might be an unrevealed outcome of 1-kestose administration.

Our findings raise questions that should be addressed in the future, such as determining a causative relationship between 1-kestose-induced changes in the gut microbiota and mitigation of glucose metabolism by adopting fecal microbiota transplantation in germ-free or antibiotic-treated mice. Thereby, we may identify mechanisms for the direct or indirect actions of GLP-1 and SCFAs on glucose metabolism. Knowledge of the interventions on glucose intolerance of T2D might be valuable for the preventative use of 1-kestose for metabolic disorders.

## Methods

### Experimental animals

All procedures for animal experiments in the present study were approved by the Animal Care Committee of the Graduate School of Bioagricultural Sciences, Nagoya University, and were carried out in accordance with relevant guidelines and regulations. Six-week-old, male OLETF rats (n = 15) and the control, Long-Evans Tokushima Otsuka (LETO) rats (n = 10) (Hoshino Laboratory Animal, Inc, Bando, Ibaraki, Japan) were obtained and individually housed in wire-mesh cages in a conventional animal room, under the conditions of controlled temperature (23 ± 1 °C) and 12-h light–dark cycle (lights on at 08:00).

### Experimental design

1-Kestose (purification > 98%) was provided by B Food Science Co., Ltd. (Chita, Aichi, Japan). After acclimatization to the animal room for about one week, the two different phenotypes of animals were randomly divided into two groups (n = 5–8 per group): the control diet (CON) group based on the AIN-93G diet and the 1-kestose diet (KES) group which was prepared by replacing sucrose in the diet with the same amount of 1-kestose at 5% (Table S1). All experimental diets were prepared in pellet form by CREA Japan (Tokyo, Japan). The experimental diets and tap water were supplied to the corresponding animals ad libitum. Food intake and body weight were recorded weekly. Water intake was recorded at age 22–23 weeks (15th–16th week of the experiment). At 14, 18, and 22 weeks of age (7th, 11th, and 15th week of the experiment, respectively), blood was collected from the tip of the tail following 8-h fasting (starting at 08:00) for measurement of plasma glucose, insulin, and C-peptide concentrations (see below). From age 20 to 21 weeks (13th to 14th week of the experiment), oral glucose tolerance tests (see below) were conducted. At age 23 weeks (the 16th week of the experiment), rats without dietary restriction were sacrificed under anesthesia with isoflurane during 10:00–12:00 on the final day, and blood samples were obtained from the posterior vena cava with a syringe to prepare serum and post-heparin plasma. Subsequently, ceca and cecal contents were collected, and pH of the cecal contents was immediately measured. Ceca and cecal contents were stored at − 80 °C until analysis.

### Measurement of fasting plasma glucose, insulin, and C-peptide concentrations

Plasma glucose was determined by the Glucose CII test WAKO (WAKO, Osaka, Japan), plasma insulin by the Rat Insulin ELISA Kit (TMB) (Shibayagi, Gunma, Japan), and plasma C-peptide by the LBIS Rat C-peptide (U-type) ELISA Kit (FUJIFILM Wako Shibayagi, Gunma, Japan).

### Oral glucose tolerance test (OGTT)

Rats were fasted for 12 h (starting from 22:00 on the day before the experiment) by removing food from the cage. At about 10:00 on the day of the experiment, blood was collected from the tip of the tail to determine the base level of plasma glucose. Then rats were administered a 20% (w/v) glucose solution at 2 g glucose/kg body weight, and blood was collected as described above at 30, 60, 90, and 120 min after administration for measurement of glucose concentration.

### Measurement of short-chain fatty acids (SCFAs) and blood components

Measurements of SCFAs (acetate, butyrate, propionate, valerate, isobutyrate, and isovalerate) were conducted as described previously^9^.

Measurements of serum total cholesterol (TC), low-density lipoprotein (LDL), high-density lipoprotein (HDL), triglyceride (TG), free fatty acids (FFA), plasma glucose, and insulin concentrations in the blood samples collected on the final day of the experiment were conducted by SRL Inc. (Tokyo, Japan).

### V3-V4-16S rRNA gene sequencing of rat cecal contents

DNA was isolated from rat cecal samples by bead-beating the sample with zirconia beads using a FastPrep FP100A instrument (MP Biomedicals, Irvine, CA) in extraction buffer (4 M guanidium thiocyanate, 100 mM Tris–HCl (pH 9.0), 40 mM EDTA), followed by extraction of the bead-treated suspension using a Magtration System 12GC and GC series MagDEA DNA 200 (Precision System Science, Matsudo, Chiba, Japan) as previously described^25^. PCR amplification of the V3-V4 region of bacterial 16S rRNA genes was conducted as previously described^25–28^. Amplicons with sample-specific barcodes were pooled for multiplex sequencing using a MiSeq Instrument (Illumina Inc., San Diego, CA), using version 3 reagent chemistry, 600 cycles, pair-end, 2 × 284-bp cycle according to a previously described method with modifications^25^. Sequences with a distance-based similarity of 99% or greater were grouped into operational taxonomic units (OTUs). Chimeric sequences that had been detected by Usearch6.1.544_i86 were precluded^29^. Based on the sequences, species were identified using the Metagenome@KIN analysis software (World Fusion, Tokyo, Japan) and the TechnoSuruga Lab Microbial Identification database DB-BA 10 (TechnoSuruga Laboratory, Shizuoka, Japan)^28,30^.

### Statistical analyses

The data were expressed as the mean ± SEM or as a scatter dot plot with a horizontal line at the median with the interquartile range. To determine differences between groups in Figs. 1, 2, 3, 4, 5 and Tables 1, 2, 3, 4, Tukey–Kramer’s test was used. Differences between groups in Fig. 6 and Table 5 were evaluated by the nonparametric Kruskal–Wallis test with Dunn’s multiple comparison test. The differences were considered significant at P < 0.05. The analyses were performed using Prism (Prism 8.2.1) software (GraphPad Software, San Diego, CA).

## Supplementary information

Supplementary file1

## References

[1] Saeedi P (2019). Global and regional diabetes prevalence estimates for 2019 and projections for 2030 and 2045: results from the International Diabetes Federation Diabetes Atlas, 9th edition. Diabetes Res. Clin. Pract..

[2] Khan MT, Nieuwdorp M, Backhed F (2014). Microbial modulation of insulin sensitivity. Cell Metab..

[3] Larsen N (2010). Gut microbiota in human adults with type 2 diabetes differs from non-diabetic adults. PLoS ONE.

[4] Qin JJ (2012). A metagenome-wide association study of gut microbiota in type 2 diabetes. Nature.

[5] Karlsson FH (2013). Gut metagenome in European women with normal, impaired and diabetic glucose control. Nature.

[6] Zhao LP (2018). Gut bacteria selectively promoted by dietary fibers alleviate type 2 diabetes. Science.

[7] Gibson GR, Roberfroid MB (1995). Dietary modulation of the human colonic microbiota: introducing the concept of prebiotics. J. Nutr..

[8] Cani PD (2007). Selective increases of bifidobacteria in gut microflora improve high-fat-diet-induced diabetes in mice through a mechanism associated with endotoxaemia. Diabetologia.

[9] Tochio T (2016). An alteration in the cecal microbiota composition by feeding of 1-kestose results in a marked increase in the cecal butyrate content in rats. PLoS ONE.

[10] Ose R (2018). The ability of human intestinal anaerobes to metabolize different oligosaccharides: Novel means for microbiota modulation?. Anaerobe.

[11] Kawano K, Hirashima T, Mori S, Natori T (1994). Oletf (Otsuka long-Evans Tokushima fatty) rat: a new NIDDM rat strain. Diabetes Res. Clin. Pract..

[12] Leighton E, Sainsbury CA, Jones GC (2017). A practical review of C-peptide testing in diabetes. Diabetes Ther.

[13] Everard A (2011). Responses of gut microbiota and glucose and lipid metabolism to prebiotics in genetic obese and diet-induced leptin-resistant mice. Diabetes.

[14] Wu T (2013). Effects of a D-xylose preload with or without sitagliptin on gastric emptying, glucagon-like peptide-1, and postprandial glycemia in type 2 diabetes. Diabetes Care.

[15] Hira T, Suto R, Kishimoto Y, Kanahori S, Hara H (2018). Resistant maltodextrin or fructooligosaccharides promotes GLP-1 production in male rats fed a high-fat and high-sucrose diet, and partially reduces energy intake and adiposity. Eur. J. Nutr..

[16] Tolhurst G (2012). Short-chain fatty acids stimulate glucagon-like peptide-1 secretion via the G-protein-coupled receptor FFAR2. Diabetes.

[17] Cummings JH, Pomare EW, Branch WJ, Naylor CPE, Macfarlane GT (1987). short chain fatty-acids in human large-intestine, portal hepatic and venous-blood. Gut.

[18] Walker AW, Duncan SH, McWilliam Leitch EC, Child MW, Flint HJ (2005). pH and peptide supply can radically alter bacterial populations and short-chain fatty acid ratios within microbial communities from the human colon. Appl. Environ. Microbiol..

[19] Chung WS (2016). Modulation of the human gut microbiota by dietary fibres occurs at the species level. BMC Biol..

[20] Duncan SH, Louis P, Flint HJ (2004). Lactate-utilizing bacteria, isolated from human feces, that produce butyrate as a major fermentation product. Appl. Environ. Microbiol..

[21] Sato T (2008). Isolation of lactate-utilizing butyrate-producing bacteria from human feces and in vivo administration of Anaerostipes caccae strain L2 and galacto-oligosaccharides in a rat model. FEMS Microbiol. Ecol..

[22] Kanauchi O (1999). Increased growth of Bifidobacterium and Eubacterium by germinated barley foodstuff, accompanied by enhanced butyrate production in healthy volunteers. Int. J. Mol. Med..

[23] Tanno H (2019). Characterization of fructooligosaccharide-degrading enzymes in human commensal Bifidobacterium longum and Anaerostipes caccae. Biochem. Biophys. Res. Commun..

[24] Feehley T (2019). Healthy infants harbor intestinal bacteria that protect against food allergy. Nat. Med..

[25] Takahashi S, Tomita J, Nishioka K, Hisada T, Nishijima M (2014). Development of a prokaryotic universal primer for simultaneous analysis of Bacteria and Archaea using next-generation sequencing. PLoS ONE.

[26] Caporaso JG (2011). Global patterns of 16S rRNA diversity at a depth of millions of sequences per sample. Proc. Natl. Acad. Sci. USA.

[27] Muyzer G, de Waal EC, Uitterlinden AG (1993). Profiling of complex microbial populations by denaturing gradient gel electrophoresis analysis of polymerase chain reaction-amplified genes coding for 16S rRNA. Appl. Environ. Microbiol..

[28] Hisada T, Endoh K, Kuriki K (2015). Inter- and intra-individual variations in seasonal and daily stabilities of the human gut microbiota in Japanese. Arch Microbiol..

[29] Edgar RC, Haas BJ, Clemente JC, Quince C, Knight R (2011). UCHIME improves sensitivity and speed of chimera detection. Bioinformatics.

[30] Kasai C (2015). Comparison of the gut microbiota composition between obese and non-obese individuals in a Japanese population, as analyzed by terminal restriction fragment length polymorphism and next-generation sequencing. BMC Gastroenterol..

