# Supplementary material for: 1-Kestose supplementation mitigates the progressive deterioration of glucose metabolism in type 2 diabetes OLETF rats
Source: Sci Rep. 2020 Sep 24;10:15674. doi: 10.1038/s41598-020-72773-2 (PMC7515885; doi:10.1038/s41598-020-72773-2)
Supplement: Supplementary file 1 — Supplementary file1 [file 41598_2020_72773_MOESM1_ESM.docx]

**Supplementary Information**

**1-Kestose supplementation mitigates the progressive deterioration of glucose metabolism in type 2 diabetes OLETF rats**

Ayako Watanabe^1,¶^, Yoshihiro Kadota^2, ¶^, Rina Kamio^1,¶^, Takumi Tochio^2^, Akihito Endo^3^, Yoshiharu Shimomura^4^, Yasuyuki Kitaura^1,^*

^1^ Laboratory of Nutritional Biochemistry, Department of Applied Biosciences, Graduate School of Bioagricultural Sciences, Nagoya University, Nagoya, Aichi, Japan

^2^ B Food Science Co., Ltd., Chita, Aichi, Japan

^3^ Department of Food, Aroma and Cosmetic Chemistry, Faculty of Bioindustry, Tokyo University of Agriculture, Abashiri, Hokkaido, Japan

^4^ Department of Food and Nutritional Sciences, College of Bioscience and Biotechnology, Chubu University, Kasugai, Aichi, Japan

Table S1. Ingredients of experimental diets

| Ingredient | Control diet (0% 1-kestose) | 1-Kestose diet (5% 1-ketose) |
| --- | --- | --- |
|  | (g/ 100g diet) | |
| Corn starch | 51.9486 | 51.9486 |
| α-Corn starch | 1.0 | 1.0 |
| Sucrose | 10.0 | 5.0 |
| Casein | 20.0 | 20.0 |
| Soybean oil | 7.0 | 7.0 |
| Cellulose | 5.0 | 5.0 |
| Mineral mix | 3.5 | 3.5 |
| Vitamin mix | 1.0 | 1.0 |
| L-Cystine | 0.3 | 0.3 |
| Choline bitartrate | 0.25 | 0.25 |
| Tert-butylhydroquinone | 0.0014 | 0.0014 |
| 1-Kestose | 0 | 5.0 |
| Total | 100.0 | 100.0 |
